# Supporting Aging-in-Place: An Innovative Program for Older Adults Living in Subsidized Housing

**DOI:** 10.1093/geroni/igaf122.1781

**Published:** 2025-12-31

**Authors:** Uri Amir-Koren, Jasmine Akman, Karen Zurlo, Ayse Akincigil

**Affiliations:** Rutgers University, New Brunswick, New Jersey, United States; Rutgers University, New Brunswick, New Jersey, United States; Rutgers University, New Brunswick, New Jersey, United States; Rutgers University, New Brunswick, New Jersey, United States

## Abstract

Traditional Assisted Living Facilities (ALFs) remain financially out of reach for many low-income older adults. This study examines an innovative assisted living model that was developed in NJ, which decouples supportive services from residential facilities, allowing seniors to receive personal care, health monitoring, and social services while remaining in subsidized public housing. By leveraging existing public housing infrastructure, this approach provides a cost-effective alternative to institutional care, promoting aging in place while maintaining access to essential services. This study utilizes a SWOT (Strengths, Weaknesses, Opportunities, and Threats) analysis to evaluate the model’s effectiveness, feasibility, and scalability. Findings indicate the program’s strengths include affordability, flexibility, and resident autonomy, while the weaknesses highlight challenges related to care coordination, funding sustainability, and workforce capacity. The opportunities for expansion are significant given the policy environment unique to the state. However, threats such as lack of price control, reimbursement challenges, labor costs and workforce dynamics, and regulatory challenges must be addressed to ensure long-term success. By systematically evaluating this model, this paper contributes to the growing body of research on alternative long-term care strategies and provides policy and practice recommendations to enhance its implementation. Expanding this model has the potential to reshape aging policy, reduce institutionalization rates, and improve quality of life for low-income older adults, making assisted living more accessible and sustainable.

